# Proteome analysis of CD5-positive diffuse large B cell lymphoma FFPE tissue reveals downregulation of DDX3X, DNAJB1, and B cell receptor signaling pathway proteins including BTK and Immunoglobulins

**DOI:** 10.1186/s12014-023-09422-z

**Published:** 2023-09-13

**Authors:** Takuya Hiratsuka, Shinji Ito, Rika Sakai, Tomoyuki Yokose, Tatsuya Endo, Yataro Daigo, Yohei Miyagi, Tatsuaki Tsuruyama

**Affiliations:** 1 Department of Drug Discovery Medicine, Pathology Division, Kyoto University Graduate School of Medicine, Kyoto, Japan; 2 Medical Research Support Center, Graduate School of Medicine, Kyoto University, Kyoto, Japan; 3 Department of Oncology, Kanagawa Cancer Center Research Institute, Yokohama, Japan; 4 Department of Pathology, Kanagawa Cancer Center Research Institute, Yokohama, Japan; 5 Department of Physics, Graduate School of Science, Tohoku University, Sendai, Japan; 6 Department of Medical Oncology, Cancer Center, and Center for Advanced Medicine Against Cancer, Shiga University of Medical Science, Otsu, Japan; 7 Center for Antibody and Vaccine Therapy, Research Hospital, Institute of Medical Science, University of Tokyo, Tokyo, Japan; 8 Molecular Pathology and Genetics Division, Kanagawa Cancer Center Research Institute, Yokohama, Japan; 9 Tazuke-Kofukai Medical Institute Kitano Hospital, Ogimachi, Osaka Japan

**Keywords:** Biomarkers, Bruton tyrosine kinase, CD5-positive Diffuse large B cell lymphoma, DNAJB1, DDX3X, Formalin-fixed paraffin-embedded specimen, Immunoglobulin, Proteomics

## Abstract

**Background:**

The molecular pathology of diffuse large B cell lymphoma (DLBCL) has been extensively studied. Among DLBCL subtypes, the prognosis of CD5-positive DLBCL is worse than that of CD5-negative DLBCL, considering the central nervous system relapse and poor response to R-CHOP therapy. However, the molecular mechanisms underlying the tumorigenesis and progression of CD5-positive DLBCL remain unknown.

**Methods:**

To identify molecular markers that can be targeted for treating DLBCL, a proteomic study was performed using liquid chromatography-mass spectrometry with chemically pretreated formalin-fixed paraffin-embedded specimens from CD5-positive (*n* = 5) and CD5-negative DLBCL patients (*n* = 6).

**Results:**

Twenty-one proteins showed significant downregulation in CD5-positive DLBCL compared to CD5-negative DLBCL. Principal component analysis of protein expression profiling in CD5-positive and CD5-negative DLBCL revealed that DNAJB1, DDX3X, and BTK, which is one of the B cell phenotypic proteins, were the most significantly downregulated proteins and served as biomarkers that distinguished both groups. Additionally, a set of immunoglobulins, including IgG4, exhibited significant downregulation. Immunohistochemistry analysis for BTK demonstrated reduced staining in CD5-positive DLBCL compared to CD5-negative DLBCL.

**Conclusions:**

In conclusion, DNAJB1 and DDX3X, BTK, and a set of immunoglobulins are promising biomarkers. Probably, the suppression of BCR signaling is the unique phenotype of CD5-positive DLBCL. This formalin-fixed paraffin-embedded (FFPE)-based profiling may help to develop novel therapeutic molecularly targeted drugs for treating DLBCL.

**Supplementary Information:**

The online version contains supplementary material available at 10.1186/s12014-023-09422-z.

## Background

Diffuse large B cell lymphoma (DLBCL) is a heterogeneous group of non-Hodgkin lymphomas exhibiting a wide variety of pathologies morphologically defined by characteristic diffuse proliferation in large B cell-derived tumor cells [1–3]. According to the 2016 World Health Organization classification [4], there are several variants or subgroups of DLBCL, with 5–10% of these are being positive for CD5, a pan-T cell marker [5]. The CD5-positive subtype is an activated B cell type of DLBCL (ABC-DLBCL), consistent with previous data from immunohistochemical arrays and comparative genomic hybridization analyses undertaken to examine gene expression profiles [6]. The CD5 antigen is a glycoprotein that modulates NF-κB signaling during development and peripheral activation [7, 8]. Additionally, CD5-positive B cells (B-1a cells) differ from other normal B cells and are derived ectopically (bone marrow independent) from organs, such as the spleen and fetal liver [9]. This B-1a subset has the potential to secrete natural antibodies, including low-affinity-binding autoantibodies, which may be related to systemic lupus erythematosus [10]. However, the specific signaling pathways associated with the poor prognosis and tumor microenvironment of CD5-positive DLBCL remain widely unclear. According to a previous genetic study, 19q13 aberration predicted a poor prognosis in non-germinal center-type CD5-positive DLBCL [11].

The use of rituximab for treating CD5-negative DLBCL has led to an improved prognosis. However, the 5-year survival rate of patients with CD5-positive DLBCL remains low [5]. Furthermore, the central nervous system recurrence rate for CD5-positive DLBCL is higher than that of CD5-negative DLBCL (CD5-positive: 12.7%, CD5-negative: 5%) [12–14]. To develop an effective first-line treatment for CD5-positive DLBCL, several studies have examined treatments that combine dose-adjusted EPOCH-R, an improvement over conventional R-CHOP, with high-dose methotrexate therapy preventing central nervous system invasion; this research includes an ongoing institutional study (PEARL) in Japan [15].

To diagnose and subclassify CD5-positive DLBCL cases, archived formalin-fixed paraffin-embedded (FFPE) specimens were used to examine the expression of known proteins using immunostaining. However, this method is limited to the identification of novel biomarkers. Recently, mass spectrometry (MS) has been applied for the analyzing FFPE samples, and a platform for MS has been established [16–21]. The present study used the FFPE-MS approach to identify upregulation or downregulation of proteins in CD5-positive DLBCL. Understanding the expression of specific proteins in CD5-positive DLBCL may help to clarify its pathology and pave the way for developing novel molecular targeted therapies.

## Methods

### Patients

Of the patients who participated in the cohort study (Grant-in-Aid for Scientific Research on Innovative Areas—Platforms for Advanced Technologies and Research Resources (16H06277)), five were selected as CD5-positive ABC-DLBCL, and six were CD5-negative ABC-DLBCL cases based on the results of immunostaining for CD10, BCL6, and MUM1 (multiple myeloma oncogene 1)/IRF4 (interferon regulatory factor 4). All samples were biopsied for diagnosis, with the patient profiles listed in Table 1.

**Table 1:** Profiles of patients with DLBCL

Patient ID	Sex	Age	CD5	CD10	Bcl6	MUM1	Bcl2	Biopsy/Operation	Organ	Phenotype of DLBCL	Stage
CD5-0	F	80 s	−	−	+	+	−	Biopsy	Neck	ABC	IA
CD5-1	M	70 s	−	−	+	+	−	Biopsy	Connective tissue	ABC	IIA
CD5-2	M	50 s	−	−	+	+	Weakly positive	Biopsy	Testis, left	ABC	IIB
CD5-3	F	50 s	−	−	+	+	−	Biopsy	Uterus	ABC	IVA
CD5-4	M	80 s	−	−	+	+	Weakly positive	Biopsy/operation	Tonsil	ABC	IIA^a^
CD5-5	M	60 s	−	−	+	+	Weakly positive	Biopsy	Pleura	ABC	IIIA
CD5-6	M	70 s	+	−	+	+	+	Biopsy	Connective tissue	ABC	IIIB
CD5-7	M	50 s	+	−	+	+	+	Biopsy	Connective tissue	ABC	IIIB
CD5-8	M	50 s	+	−	+	+	+	Biopsy	Connective tissue	ABC	IVB
CD5-9	F	60 s	+	−	+	+	+	Biopsy	Oral cavity/ pharynx	ABC	IIA
CD5-10	F	50 s	+	−	+	+	+	Biopsy	Connective tissue	ABC	IVA

*ABC* activated B cell type

^a^DLBCL with Philadelphia chromosome-positive acute lymphocytic leukemia

### Tissue preparation

After fixation using 10% neutral-buffered 4% (v/v) formaldehyde solution, the tissues were embedded in paraffin. The paraffin-embedded blocks were then sliced into 4 μm sections on glass slides for subsequent microscopic observation using an Olympus BX43 microscope after hematoxylin and eosin staining. After observation, additional 10-μm sections were prepared on glass slides for proteomic analysis collected in a 2-mm tube. FFPE blocks were prepared using Tissue-Tek® VIP® 6 (Sakura Finetech Japan, Tokyo, Japan).

### Protein extraction

Proteins were extracted using a previously described method, with some modifications [16, 22]. The tissue on the glass slides was deparaffinized before being scraped into a 2-mL tube. Each sample was suspended in 20 µL of 0.1 mol/L NH_4_HCO_3_ (Nacalai Tesque, Kyoto, Japan) containing 30% (v/v) CH_3_CN (FUJIFILM Wako Pure Chemical Corporation, Osaka, Japan), and homogenized thrice using a lysis and homogenization system (Precellys Evolution, Bertin Technologies SAS, Montigny-le-Bretonneux France) at 7,800 rpm for 1 min (three times), and centrifuged at 10,000 ×*g* for 1 min. Next, the supernatants were heated at 95 °C for 90 min in a water bath. The supernatants were vortexed and centrifuged using a mini centrifuge (Nippon Genetics, Tokyo) every 30 min. They were centrifuged again at 10,000 × *g* for 1 min and chilled on ice, followed by the addition of 1 µg of trypsin (Promega, Madison, WI) and 2 µg of lysyl endopeptidase, MS Grade (FUJIFILM Wako Pure Chemical Corporation, Osaka, Japan). Samples were incubated at 37 °C for 16 h. The proteins were purified and concentrated for MS using Pierce™ C18 Spin Columns (Thermo Scientific, Cramlington, UK). The protein concentration was estimated using the bicinchoninic acid method (BCA Assay; Thermo Scientific, Cramlington, UK); the proteins were then separated by sodium dodecyl sulfate–polyacrylamide gel electrophoresis and stained with Coomassie Brilliant Blue.

### LC/MS

Protein samples were separated using Nano-LC Ultra 2D-plus equipped with cHiPLC Nanoflex (Eksigent, Dublin, CA) in the trap-and-elute mode, using a trap column (200 μm × 0.5 mm ChromXP C18-CL 3 μm 120 Å (Eksigent)) and an analytical column (75 μm × 15 cm ChromXP C18-CL 3 μm 120 Å (Eksigent)). The separation was carried out using a binary gradient in which 0.1% formic acid/water and 0.1% formic acid/acetonitrile were used as solvents A and B, respectively. The gradient program was as follows: 2 to 33.2% B for 125 min, 33.2 to 98% B in 2 min, 98% B for 5 min, 98 to 2% B in 0.1 min, and 2% B for 17.9 min. The flow rate was 300 nL/min. The analytical column temperature was set to 40 °C. The eluates were infused on-line to the mass spectrometer, TripleTOF 5600 + System with NanoSpray III source, and a heated interface (SCIEX, Framingham, MA) and then ionized using an electrospray ionization-positive mode. Data acquisition was carried out with an information-dependent acquisition method. The acquired datasets were analyzed using the ProteinPilot software version 5.0.1 (SCIEX, Framingham, MA, USA) with the UniProtKB/Swiss-Prot database for humans (May 2018) appended with known common contaminant database (SCIEX). Paragon algorithm (SCIEX) was used for the protein identification including false discovery rate analysis with the detection threshold set to ProtScore &gt; 0.05 (Confidence &gt; 10%). No user modifications were made on the XML files stored in the ProteinPilot workflow directory. We also provided raw data generated by ProteinPilot and Progenesis QI for Proteomics software (Additional file 1). The relative abundances of the identified proteins were estimated on the platform, Progenesis QI platform of Proteomics software version 4.1 (Nonlinear Dynamics, Newcastle upon Tyne, UK). All raw data files were imported to generate an aggregate file, and the peptide identification results obtained by the ProteinPilot software, with confidence levels of at least 95%, were used for assignment. Label-free quantification of proteins was performed by relative quantitation using the Hi-N(3) method (Nonlinear Dynamics, Newcastle upon Tyne, UK).

### Immunohistochemistry (IHC)

FFPE sections of DLBCL were immunostained and subsequently visualized using 3,3′-diaminobenzidine. Counterstaining was performed with Mayer’s hematoxylin. DLBCL tissues were obtained as a tissue microarray [(core size, 1.0 mm), LY1001d, LY2085, US Biomax, Inc., Rockville, MD]. The antibodies used are listed in Additional file 3: Table S1.

### Gene ontology and functional enrichment

For the pre-processing of proteins for Gene Ontology, each Protein ID was converted to its generic gene name using UniProt. Gene Ontology and functional enrichment were performed using DAVID v6.8 (https://david.ncifcrf.gov/). The DAVID Gene system was built by combining the NCBI Entrez Gene database with Uniprot. The Knowledgebase includes data from PubChem, DrugBank, the Human Protein Atlas, DisGeNET, WikiPathways, and PathBank. Uniprot annotation included eight subgroups with the original “"Functional”" category and Disease, Protein interactions, and Tissue Expression. The results of the Gene Ontology analysis were categorized into Biological Process (BP), Molecular Functions (MF), and Cellular Component (CC). The enriched pathways were then subjected to the Kyoto Encyclopedia of Genes and Genomes (KEGG) and Reactome analyses. Results with p-values ≤ 0.01 were considered statistically significant.

### Protein–protein interaction (PPI) network reconstruction

The Search Tool for the Retrieval of Interacting Genes (STRING) (https://string-db.org/) was used to construct the PPI network. The interrelationship of identified proteins in regulating the cellular pathways was constructed using NetworkAnalyst. Experimentally validated interactions with high confidence values (0.9) were significant in this analysis.

### Statistical analysis

The statistical significance level here was set at 0.05. Meanwhile, in MS screening, *p* < 0.025 (0.05/2) was considered significant after Bonferroni’'s correction. Briefly, fold-change values > 1.5 and *p* values < 0.025 were considered significant for lymphoma tissue screening. Furthermore, results with *p* values < 0.05 were considered statistically significant when comparing the abundance of each protein in CD5-positive and CD5-negative DLBCL. The possibility of the expression level of each subset of immunoglobulins being higher or lower in CD5-positive or CD5-negative DLBCL was subjected to a test using a bimodal distribution (rejection area *p* = 0.05). Statistical calculations were performed using the SPSS software (IBM, Armonk, NY). Additionally, Pearson’s correlation analysis and principal component analysis (PCA) were conducted using Python 3.0 on Jupyter Notebook (6.3.0), an interactive computing notebook environment.

## Results

### Histopathology of DLBCL samples

**Fig. 1:**
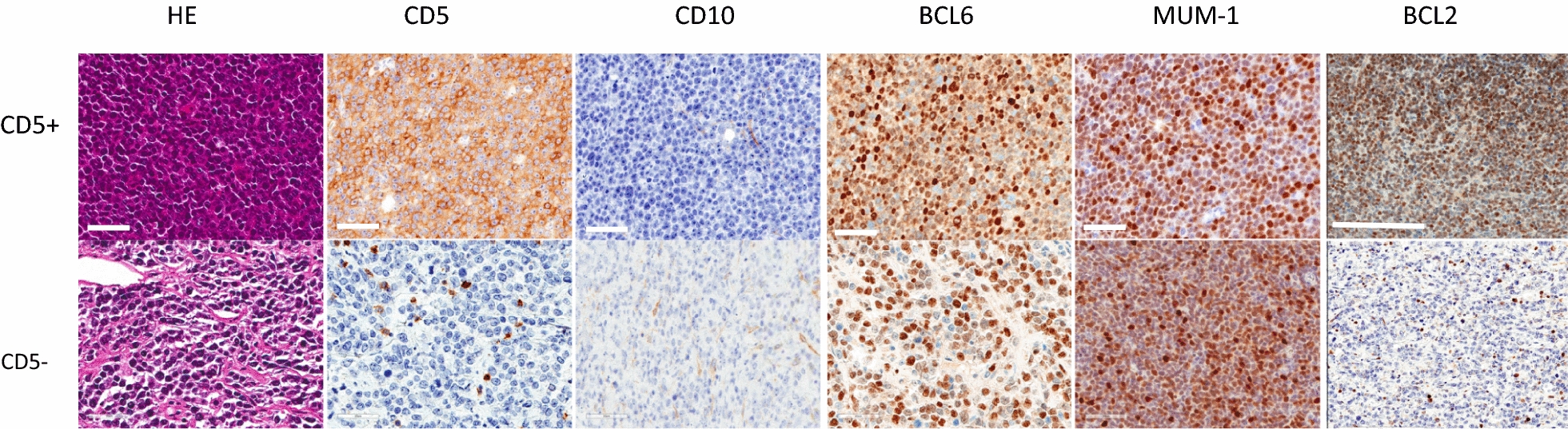
Histological examination of formalin-fixed paraffin-embedded (FFPE) specimens. Representative immunohistochemistry (IHC) images of CD5-positive (CD5-6) and CD5-negative DLBCL (CD5-3) specimens stained with hematoxylin and eosin (HE), or immunostained with antibodies against CD5, CD10, BCL6, MUM-1, and BCL2. In this case, the IHC of CD5-positive (CD5+) shows CD5 +, CD10 −, BCL6 +, MUM-1 +, and BCL2 +. CD5-negative (CD5-) DLBCL shows CD5 −, CD10 −, BCL6 +, MUM-1 +, and BCL2 −. Non-tumor cells were excluded from the positive cells. Scale bar, 50 µm in CD5, CD10, BCL6, and MUM-1 and 200 µm in BCL2

LC/MS of lymphoma tissue was performed using FFPE samples from six patients with CD5-negative DLBCL and five patients with CD5-positive DLBCL (Table 1). The histological images and data for each immunostained sample are shown in Fig. 1. All CD5-positive DLBCL samples were positive for CD5, BCL6, BCL2, and MUM-1 and negative for CD10. In contrast, CD5-negative DLBCL samples were positive for BCL6 and MUM-1, but not for CD10. Based on these immunostaining results, both CD5-positive and CD5-negative DLBCL samples were diagnosed as belonging to the ABC-DLBCL type (Table 1) [23].

### Protein identification and quantification

**Fig. 2:**
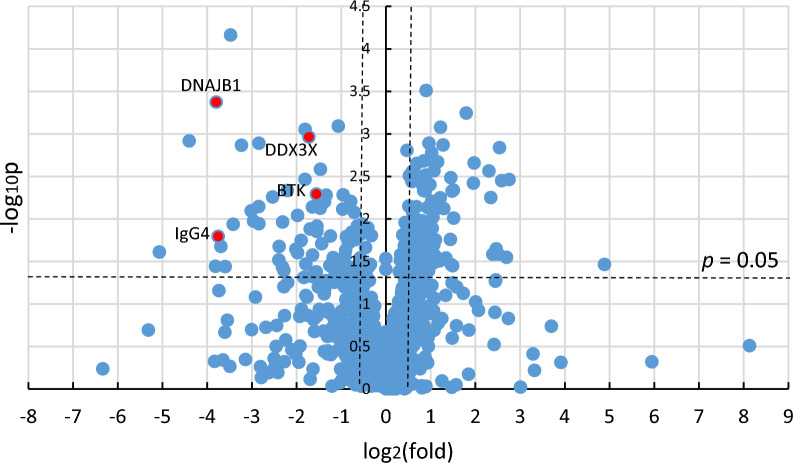
Comparison of overall protein expression in CD5-positive and CD5-negative DLBCLs. Volcano plots show the protein expression between CD5-positive (CD5+) and CD5-negative (CD5-) DLBCLs. Each point represents an individual protein. The *x*-axis shows the log2 value of the  fold change of normalized abundance (peptide amount) for two lymphomas. Positive values indicate that the proteins are larger, and negative values indicate that proteins are smaller in CD5 + DLBCL. The *y*-axis shows the negative common logarithm of the ANOVA *p*-value. The vertical dotted line represents a 1.5-fold change, and horizontal dotted lines denote *p* = 0.05 (Mann–Whitney *U* test). The selected proteins are labeled using their UniProt entry names (See Table 2)

LC–MS was performed on selected tissue samples from the patients. Overall, 1,059 proteins were identified from the obtained spectra, with a false discovery rate of < 1% (Additional file 1: Data S1). The distribution of all protein normalized abundance was illustrated using a volcano plot (Fig. 2), and each the normalized abundance of each ionized peptides in both DLBCLs was statistically compared.

The expression of 114 proteins was significantly higher in CD5-positive than CD5-negative DLBCL, while the expression of 21 proteins was significantly lower in CD5-positive than CD5-negative DLBCL. The possible functional roles of the 114 upregulated proteins in CD5-positive DLBCL were analyzed using DAVID v6.8. As a result of Gene Ontology analysis, upregulated proteins were found to be associated with translational initiation, cell–cell adhesion, and rRNA processing in BP (Additional file 2: Fig. S1a); RNA metabolism-related proteins in Molecular Function analysis (MF) (Additional file 2: Fig. S1b); and extracellular exosome in CC (Additional file 2: Fig. S1c). Moreover, interactome analysis using the STRING platform revealed constructing the protein–protein interaction network for the identification of hub proteins in upregulated proteins of CD5-positeve DLBCL (Additional file 2: Fig. S2a, b).

21 downregulated proteins were analyzed using DAVID v6.8. BP analysis subsequently revealed significant enrichment in cell–cell adhesion (Table 2, Additional file 2: Fig. S3a). Meanwhile, MF indicated that the downregulated proteins were associated with cadherin-mediated cell–cell adhesion, extracellular matrix (ECM) structural constituent, and identical protein binding (Additional file 2: Fig. S3b). CC indicated proteinaceous ECM (Additional file 2: Fig. S3c). Pearson’s sample–sample correlation analysis clearly showed a clear correlation (*R*^*2*^ > 0.90) between CD5-positive DLBCL cases; this group was found to be a much more uniform disease population than CD5-positive DLBCL (Additional file 2: Fig. S4, Additional file 3: Table S2).

**Table 2:** Biomarker candidates

Accession	Protein name	Description
P25685|DNJB1_HUMAN	DNAJB1	DnaJ homolog subfamily B member 1, a molecular chaperone that prevents misfolded protein aggregation
O00571|DDX3X_HUMAN	DDX3X	DEAD box helicase 3, X-linked, ATP-dependent RNA helicase
sp|Q06187|BTK_HUMAN	BTK	Bruton Tyrosine Kinase
Immunoglobulins		
sp|P01861|IGHG4	Heavy constant gamma 4	
sp|A0A0A0MRZ8|KVD11;sp|P04433|KV311	Kappa variable 3D-11	
sp|P01619|KV320	Kappa variable 3–20	
sp|P0DOY2|IGLC2;sp|P0DOY3|IGLC3	Lambda constant 2	
sp|P01876|IGHA1	Heavy constant alpha 1	
sp|P01591|IGJ	J chain	
sp|P01834|IGKC	Kappa constant	
sp|P01859|IGHG2	Heavy constant gamma 2	
sp|A0A0C4DH55|KVD07;sp|P01624|KV315	Kappa variable 3D−7	
sp|P0DOX5|IGG1_	Gamma-1 heavy chain	
sp|A0A075B6P5|KV228;sp|A0A087WW87|KV240;sp|P01614|KVD40;sp|P01615|KVD2	Kappa variable 2–28	

### Principal component analysis (PCA)

**Fig. 3:**
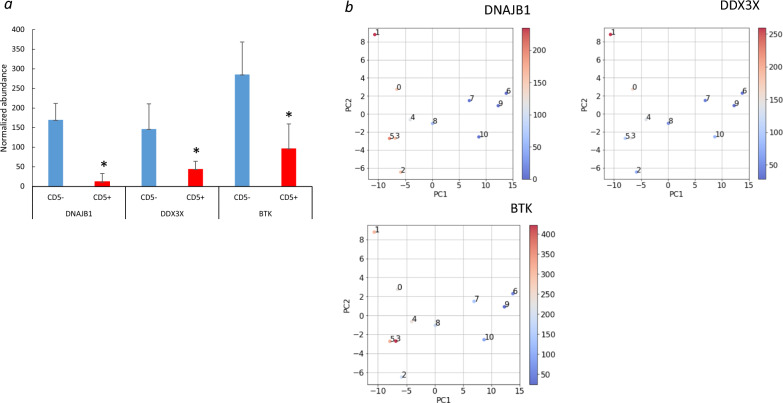
Principal component analysis. **a** Normalized abundance of DNAJB1, DDX3X, and BTK. The asterisk (*) indicates *p* < 0.05 for CD5 + *vs*. CD5- DLBCL using Student’s *t*-test. The vertical axis represents the normalized abundance of proteins. Error bars represent the standard deviation of normalized abundance. **b** Principal component analysis. The heat gradient represents the normalized abundance of DNAJB1, DDX3X, and BTK in CD5-negative DLBCL (patients 0–5) and CD5-positive DLBCL (patients 6–10)

Only three proteins, DNAJB1, DDX3X, and Bruton tyrosine kinase (BTK), were found to be decreased in all CD5-positive DLBCL cases compared to CD5-negative DLBCL cases. In particular, BTK is considered one of the key proteins determining the phenotype of DLBCL, a mature B-cell tumor. Furthermore, a Principal Component Analysis (PCA) was performed on all proteins that exhibited significant differences in normalized abundance between CD5-positive DLBCL and CD5-negative DLBCL (Fig. 3a; Additional file 3: Table S3). As a result, DNAJB1 (0.07-fold downregulated; *p* < 4.0 × 10^–5^), DDX3X (0.3-fold downregulated, *p* = 0.01), and BTK1 (0.33-fold downregulated; *p* = 0.002) (Fig. 3b, Additional file 2: Fig. S5a, b). In addition, DNAJB1 and DDX3X form a PPT network as hub proteins. The most significantly enriched pathways included cellular responses to stimuli and heat shock (Additional file 2: Fig. S6a, b). The essential functions of DNAJB1, DDX3X, and BTK are summarized in Table 2.

### BTK and a set of immunoglobulins

**Fig. 4:**
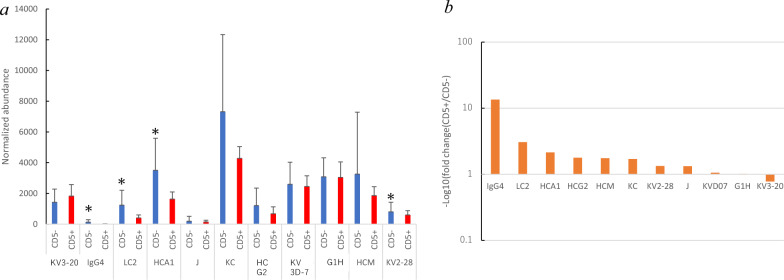
Expression of immunoglobulin, BTK. **a** The vertical axis represents the normalized abundance of each immunoglobulin (heavy constant gamma 4, IgG4; lambda constant 2, LC2; kappa variable 3D-7, KV3D-7; kappa variable 3–20, KV320; kappa variable 2–28, KV2-28; kappa constant, KC; J chain, J; heavy constant mu, HCM; heavy constant gamma 2, HCG2; heavy constant alpha 1, HCA1; gamma-1 heavy chain, G1H. Immunoglobulin kappa variable 3D-11 was excluded because only one case of CD5 + DLBCL showed significantly high levels. The asterisk (*) indicates *p* < 0.05 for CD5 + *vs*. CD5- DLBCL using Student’s *t*-test. Error bars represent the standard deviation of normalized abundance. **b** The *y*-axis shows the negative log2 value of the magnification of normalized abundance for two lymphomas. Negative values indicate that CD5 + DLBCL is highly abundant, and positive values indicate that its abundance is low

The identified BTK is a critical molecule in the signaling pathway of the immunoglobulin-forming B cell receptor (BCR). The BCR signal pathway is responsible for transmitting B cell-specific developmental signals. The BCR consists of an immunoglobulin and CD79a/b adapter protein that recruits protein kinases, such as spleen tyrosine kinase (SYK) and BTK [24, 25]. Although no significant difference was observed in the normalized abundance of individual immunoglobulin segments between CD5-positive and CD5-negative DLBCL samples, the mean abundance values for the 11 detected segments were lower in the former than the latter, including IgG4, except for Ig *κ* variable 3–20. This indicated a decreasing trend in immunoglobulin expression in the former through a binomial test (*p* < 0.05) (Fig. 4a, b). As aforementioned, the level of BTK was significantly reduced in CD5-positive tumors (Fig. 3a). PCA analysis also showed that the IgG4 and BTK abundance data contributed to distinguishing between CD5-negative and CD5-positive DLBCLs (Fig. 3b, Additional file 2: Fig. S5a).

### IHC for BTK

**Fig. 5:**
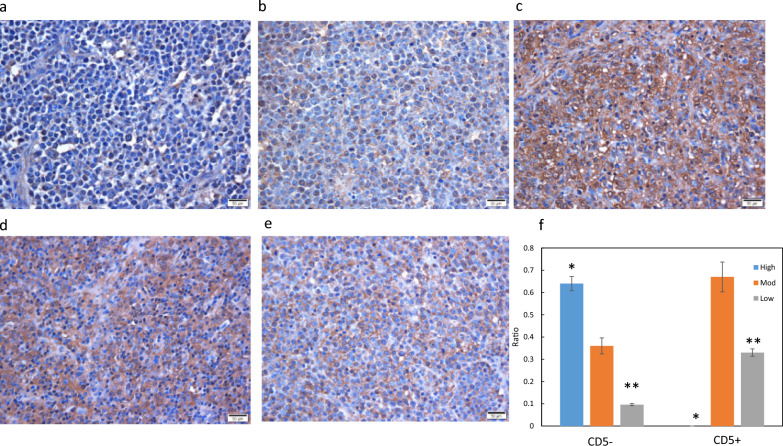
BTK immunohistochemistry in DLBCL. The photos show the representative BTK IHC images for **a** Low CD5-positive DLBCL, **b** Medium CD5-positive DLBCL, **c** High CD5-negative DLBCL, **d** Medium CD5-negative DLBCL, **e** Medium CD5-negative DLBCL, Scale bar = 50 µm. **f** The vertical axis represents the proportion of stainability, low, medium, and high. The asterisk (*) indicates p < 0.05 for CD5 + *vs*. CD5- in Student’s *t*-test. High, Mod, and Low indicate high, moderate, and low stainability using anti-BTK. The error bars indicate the standard deviation

To confirm that normalized BTK abundance was lower in CD5-positive tissues in LC/MS analysis, DLBCL tissue arrays were immunostained using anti-BTK (Fig. 5a–e; Additional file 3: Table S4). The array contained 134 DLBCL cases. Nine DLBCL cases positive for CD5 were selected using immunostaining and then subjected to IHC for BTK. Among the CD5-negative tissues (*n* = 125), 64% showed intensely diffuse positivity in the cytoplasm (High), 36% showed diffuse and medium positivity (Medium), and the remaining 9.6% showed weak and diffuse positivity for BTK (Low). In contrast, none of the nine cases of CD5-positive DLBCL showed high expression of BTK, 67% showed medium expression, and 33% showed low expression. Based on these results, the frequencies of high (*, CD5 − vs. CD5+, *p* < 0.01) and low positivity (**, CD5- *vs*. CD5+, *p* < 0.01) were significantly different between CD5-negative and CD5-positive DLBCL (Fig. 5f). In conclusion, the expression of BTK was substantially lower in CD5-positive DLBCL.

## Discussion

This study aimed to identify molecular markers for the pathological diagnosis of CD5-positive DLBCL using proteomic analysis of FFPE specimens.

The current proteomic analysis was also effective in identifying the downregulated markers. Considering that downregulated proteins are associated with ECM production and cell–cell adhesion, their decrease may be responsible for the aggressiveness and metastasis of CD5-positive DLBCL. DNAJB1 downregulation has not been addressed in lymphomagenesis, whereas DNAJB1 has been reported as one of the target genes in multiple myeloma [26]. DNAJB1 has the highest node degree distribution among the proteins detected, forming a network with a set of heat shock proteins, such as HSP90AB1, HSPH1, HSPA1B, HSPA1L, and HSPA6 (Additional file 2: Fig. S6a, b). A decrease in DNAJB1 may lead to less protein structure stabilization by chaperone proteins in interactions with HSP90AB1, HSPH1, HSPA1B, HSPA1L, and HSPA6. This profiling is opposed to that reported in multiple myeloma in which these are increased [26], indicating that CD5-positive DLBCL has an immature B cell-lineage phenotype. 

Furthermore, DDX3X loss indicates a poor prognosis for DLBCL related to the increase in STAT3/p42/p44 phosphorylation [27]. DDX3X is an ATP-dependent RNA helicase involved in various biological processes such as transcriptional regulation, cell adhesion, signal transduction, and stem cell formation. Mutations or loss of DDX3X are known in DLBCL, Burkitt’s lymphoma, cutaneous T-cell lymphoma, and NK/T-cell lymphoma [27–30]. Mutation or loss of DDX3X leads to increased expression of cyclin D1 and activation of ERK and JAK-STAT systems, increased cell proliferation and mobility, poor prognosis, and drug resistance [2, 4–7, 27, 30–33]. It is known that the Ki-67 positivity rate increases and the stage of disease progression. Abnormalities in p53 can further worsen the prognosis [30, 34]. These markers indicate a worse prognosis than CD5-negative DLBCL due to CD5-positive DLBCL and may be involved in early systemic metastasis and progression of the disease stage to refractory status [12–15].

BTK and ten immunoglobulins were found to be decreased in CD5-positive DLBCL (Figs. 2, 3a, 4a, b, 5f). Considering that BTK and immunoglobulin form BCR signaling, this comprehensive decrease suggests a downregulation of BCR signaling, which may be responsible for the immune phenotype of CD5-positive DLBCL. BTK is known to enhance cyclin D2 expression that promotes the growth of lymphoma cells [35]. While BTK inhibitors are emerging drugs that promote apoptosis in DLBCL [36], the BCR signal suppression in CD5-positive DLBCL may make these cells resistant to BTK inhibitors. If the BCR signal pathway is not the dominant driving the growth of CD5-positive DLBCL, it is plausible that an alternative signaling cascade may be contributing to their proliferation and survival. Further research is needed to elucidate the specific signaling pathways that govern the biology of CD5-positive DLBCL, which could pave the way for the development of targeted therapies tailored to this distinct subgroup of patients. Another distinctive feature was the significant decrease in IgG4. This immunoglobulin subtype is prominent in IgG4-related diseases. It has been suggested that IgG4 does not lead to the release of chemical mediators, but rather may suppress the release of mediators by competing with IgE for antigens [37]. There are not many reports of an association between IgG4-RD and DLBCL [38]. The reduction of this subtype in CD5-positive DLBCL may lead to the induction of inflammation. However, it remains uncertain whether this phenomenon is directly related to patient prognosis. Combined with the overall decline in immunoglobulin recombination, it may also be linked to immaturity preceding lymphomagenesis due to a general decline in the B cell signaling pathway.

Finally, the role of CD5 remains controversial in this study. CD5 can recruit small adaptor molecules such as a heterodimer partner (SHP). Further, Lck is involved in phosphatidylinositol 3-kinase, AKT, and other intracellular signaling pathways [38–41]. In future studies, it is necessary to clarify the relationship between the downregulation of BCR signal cascade and CD5.

## Conclusions

Overall, this study demonstrated that proteomics analysis using FFPE specimens can comprehensively reveal essential pathways involved in CD5-positive DLBCL proliferation and suggest therapeutic strategies for its treatment. In particular, our data demonstrated the suppression of B cell signals, which may help to develop novel therapeutic molecularly targeted drugs for treating DLBCL. Furthermore, analysis of archival samples using the approach applied in this study may facilitate the identification of target molecules in rare diseases.

### Supplementary Information

**Additional file 1.** Proteomics data for group 0 (CD5-negative DLBCL) and group 1(CD5-positive DLBCL).

**Additional file 2: Figure S1.** Gene ontology (GO) and Kyoto Encyclopedia of Genes and Genomes (KEGG) pathway enrichment of the most significantly upregulated proteins in CD5-positive DLBCL. Significant (Benjamini-corrected *p *< 0.01) (a) Biological processes, (b) Molecular functions, and (c) Cellular components associated with upregulated proteins in CD5-positive DLBCL based on DAVID analysis. **Figure S2.** Pathway modules involving upregulated proteins in CD5-positive DLBCL. (a) Pathway-derived networks and (b) Reactome significantly enriched (FDR < 0.01) with upregulated proteins in CD5-positive DLBCL. **Figure S3.** Gene ontology (GO) and Kyoto Encyclopedia of Genes and Genomes (KEGG) pathway enrichment of the most significantly downregulated proteins in CD5-positive DLBCL. (a) Biological processes, (b) Molecular functions, and (c) Cellular components associated with downregulated proteins in CD5-positive DLBCL based on DAVID analysis. **Figure S4.** Correlation analysis of protein intensity in CD5-negative DLBCL and CD5-negative DLBCL. Pearson’s correlation coefficient analysis of the intensity between CD5-negative (patient 1-6) and CD5-positive (patient 7-11) for the validation of processed data. All protein intensities were analyzed, and intensity values were transformed (log10). **Figure S5.** PCA analysis of upregulated and downregulated genes in CD5-positive DLBCL. (a) Principal component analysis of proteins detected in all patient FFPE tissue samples. (b) Accumulation contribution ratios in PCA. **Figure S6.** Pathway modules involving downregulated proteins in CD5-positive DLBCL. (a) Pathway-derived networks and (b) Reactome significantly enriched (FDR < 0.01) with the downregulated proteins in CD5-positive DLBCL.

**Additional file 3: Table S1.** Protocol for immunohistochemical assay of DLBCLs. **Table S2.** Correlation coefficient in patients 0–10. **Table S3**. Explained variance (Principal component contribution rate). **Table S4**. Profiling of patients of TMA.

## Data Availability

All relevant data are presented in the current manuscript or supplementary material (Additional files 1, 2 and 3). The raw data of the current study are available from the corresponding author upon reasonable request.

## Abbreviations

DLBCL: Diffuse large B cell lymphoma
MS: Mass spectrometry
FFPE: formalin-fixed paraffin embedded
BP: Biological Process
CC: Cellular Component
MF: Molecular Functions
PPI: Protein–protein interaction
BTK: Bruton tyrosine kinase
BCR: B cell receptor
