# Supplementary material for: Proteome analysis of CD5-positive diffuse large B cell lymphoma FFPE tissue reveals downregulation of DDX3X, DNAJB1, and B cell receptor signaling pathway proteins including BTK and Immunoglobulins
Source: Clin Proteomics. 2023 Sep 13;20:36. doi: 10.1186/s12014-023-09422-z (PMC10498596; doi:10.1186/s12014-023-09422-z)
Supplement: Supplementary file 2 — Additional file 2: Figure S1. Gene ontology (GO) and Kyoto Encyclopedia of Genes and Genomes (KEGG) pathway enrichment of the most significantly upregulated proteins in CD5-positive DLBCL. Significant (Benjamini-corrected p < 0.01) (a) Biological processes, (b) Molecular functions, and (c) Cellular components associated with upregulated proteins in CD5-positive DLBCL based on DAVID analysis. Figure S2. Pathway modules involving upregulated proteins in CD5-positive DLBCL. (a) Pathway-derived networks and (b) Reactome significantly enriched (FDR < 0.01) with upregulated proteins in CD5-positive DLBCL. Figure S3. Gene ontology (GO) and Kyoto Encyclopedia of Genes and Genomes (KEGG) pathway enrichment of the most significantly downregulated proteins in CD5-positive DLBCL. (a) Biological processes, (b) Molecular functions, and (c) Cellular components associated with downregulated proteins in CD5-positive DLBCL based on DAVID analysis. Figure S4. Correlation analysis of protein intensity in CD5-negative DLBCL and CD5-negative DLBCL. Pearson’s correlation coefficient analysis of the intensity between CD5-negative (patient 1-6) and CD5-positive (patient 7-11) for the validation of processed data. All protein intensities were analyzed, and intensity values were transformed (log10). Figure S5. PCA analysis of upregulated and downregulated genes in CD5-positive DLBCL. (a) Principal component analysis of proteins detected in all patient FFPE tissue samples. (b) Accumulation contribution ratios in PCA. Figure S6. Pathway modules involving downregulated proteins in CD5-positive DLBCL. (a) Pathway-derived networks and (b) Reactome significantly enriched (FDR < 0.01) with the downregulated proteins in CD5-positive DLBCL. [file 12014_2023_9422_MOESM2_ESM.pptx]

## Slide 1
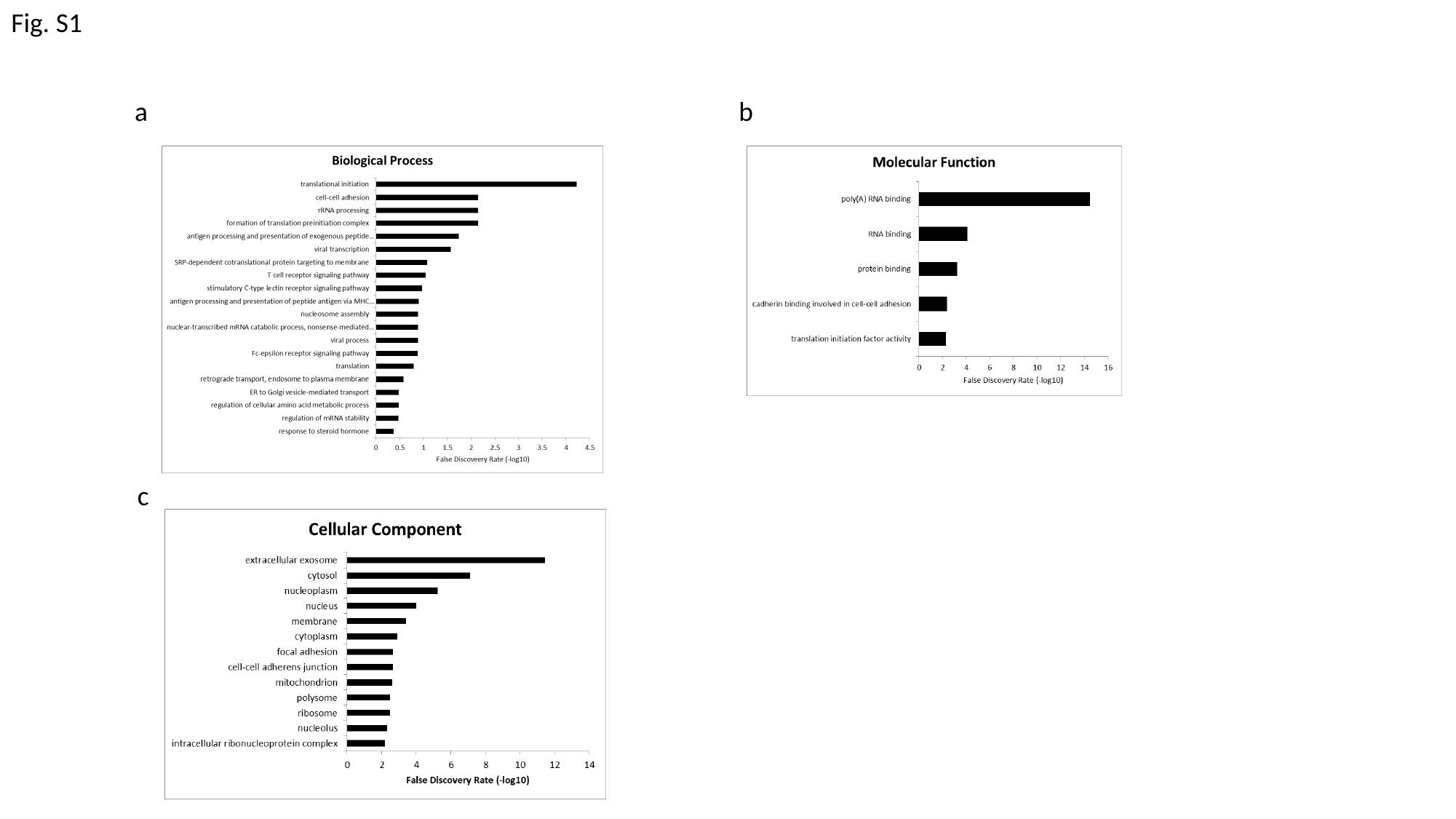

Fig. S1
a
b
c

## Slide 2
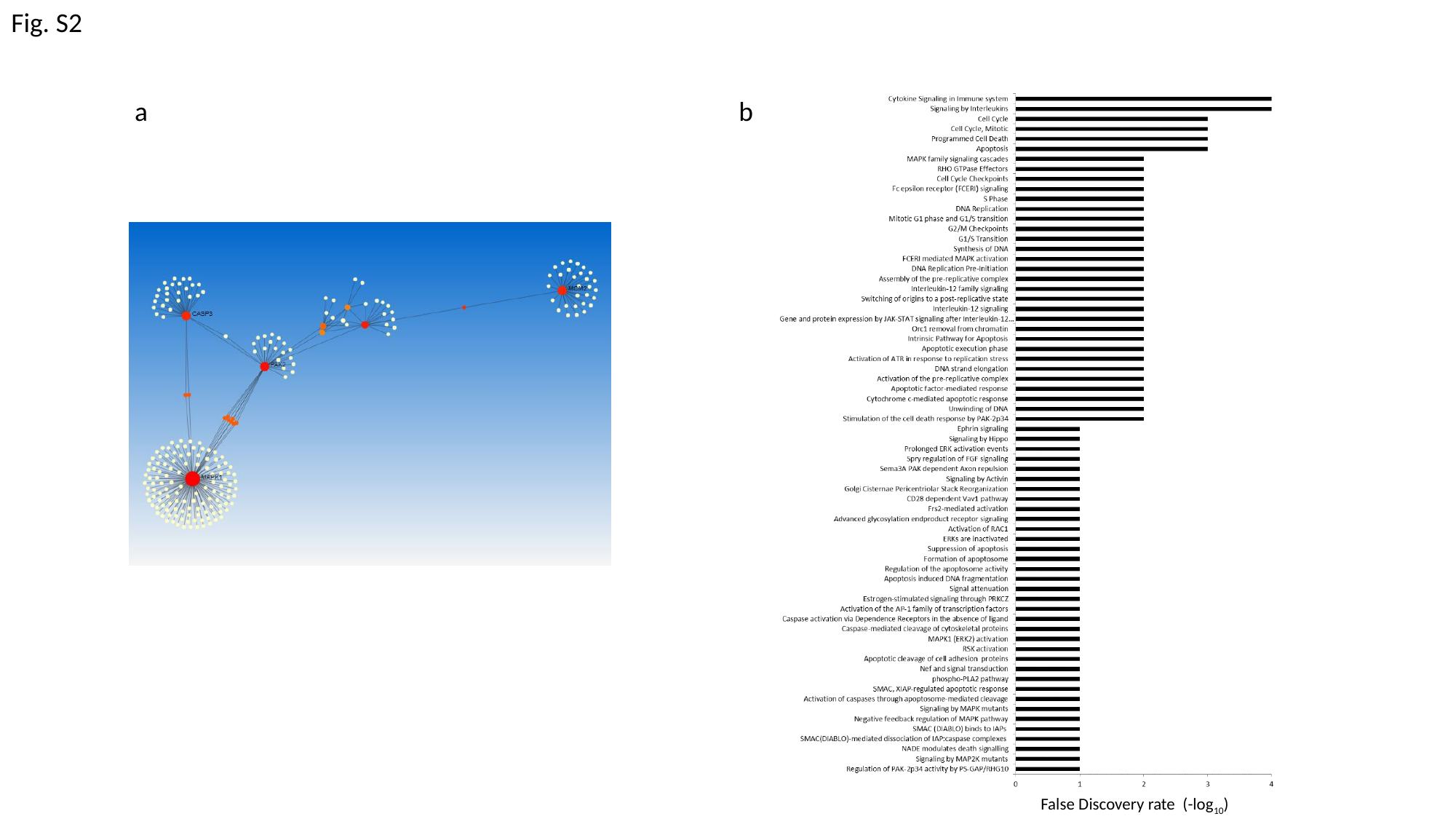

Fig. S2
a
b
False Discovery rate (-log10)

## Slide 3
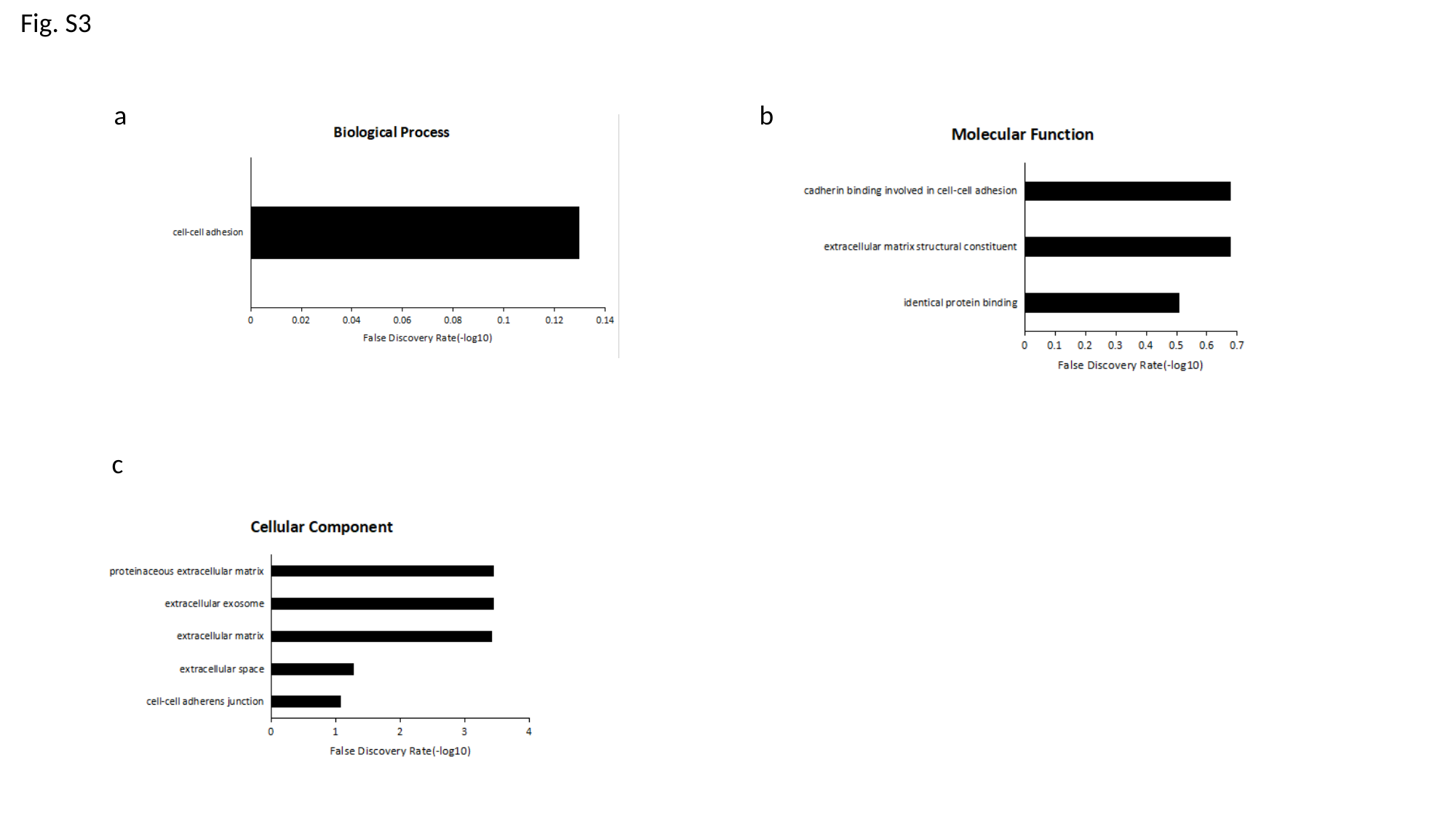

Fig. S3
a
b
c

## Slide 4
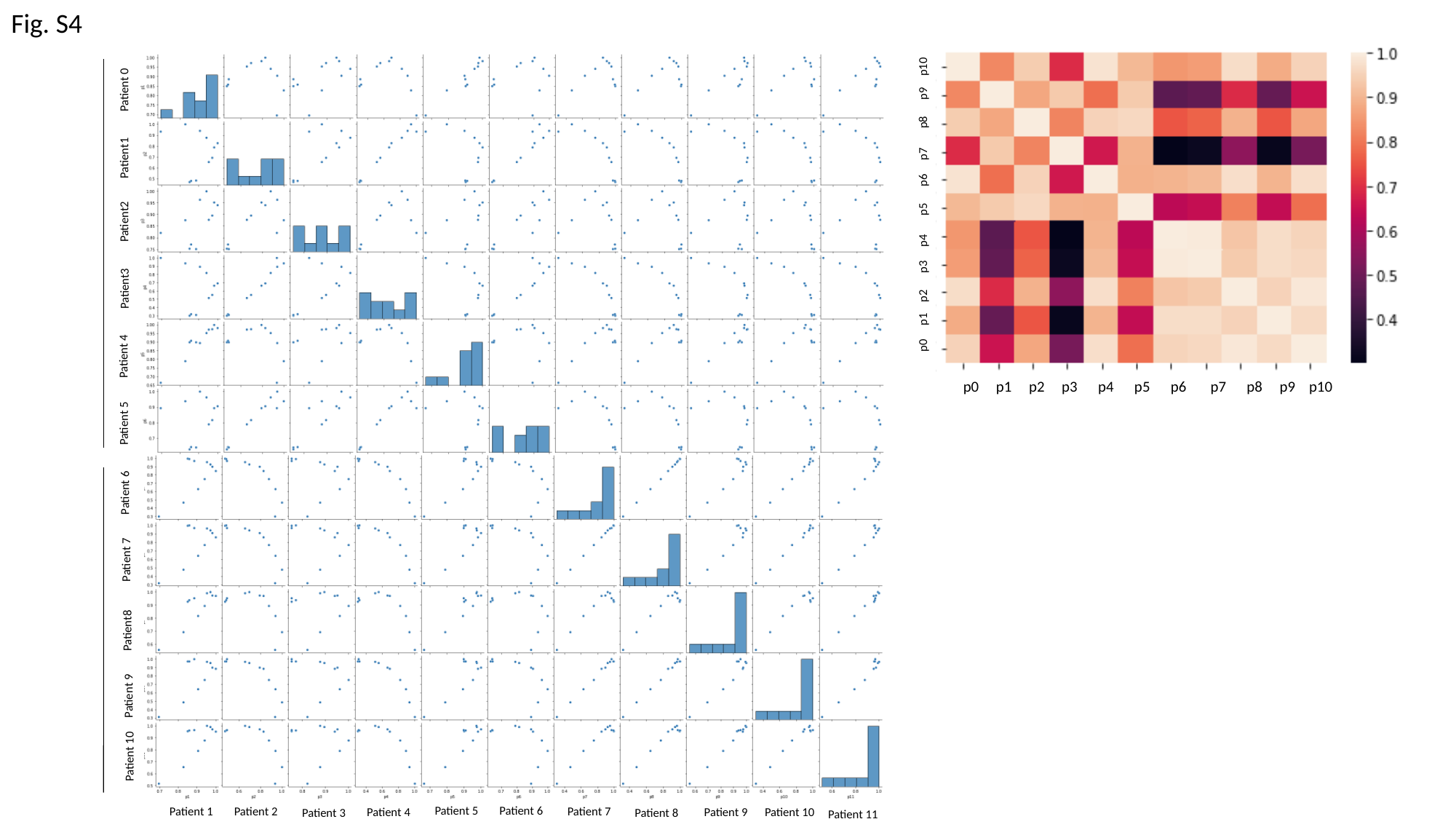

Fig. S4
Patient 0
Patient1
p0 p1 p2 p3 p4 p5 p6 p7 p8 p9 p10
Patient2
Patient3
Patient 4
p0 p1 p2 p3 p4 p5 p6 p7 p8 p9 p10
Patient 5
Patient 6
Patient 7
Patient8
Patient 9
Patient 10
Patient 6
Patient 5
Patient 7
Patient 2
Patient 1
Patient 10
Patient 9
Patient 4
Patient 3
Patient 8
Patient 11

## Slide 5
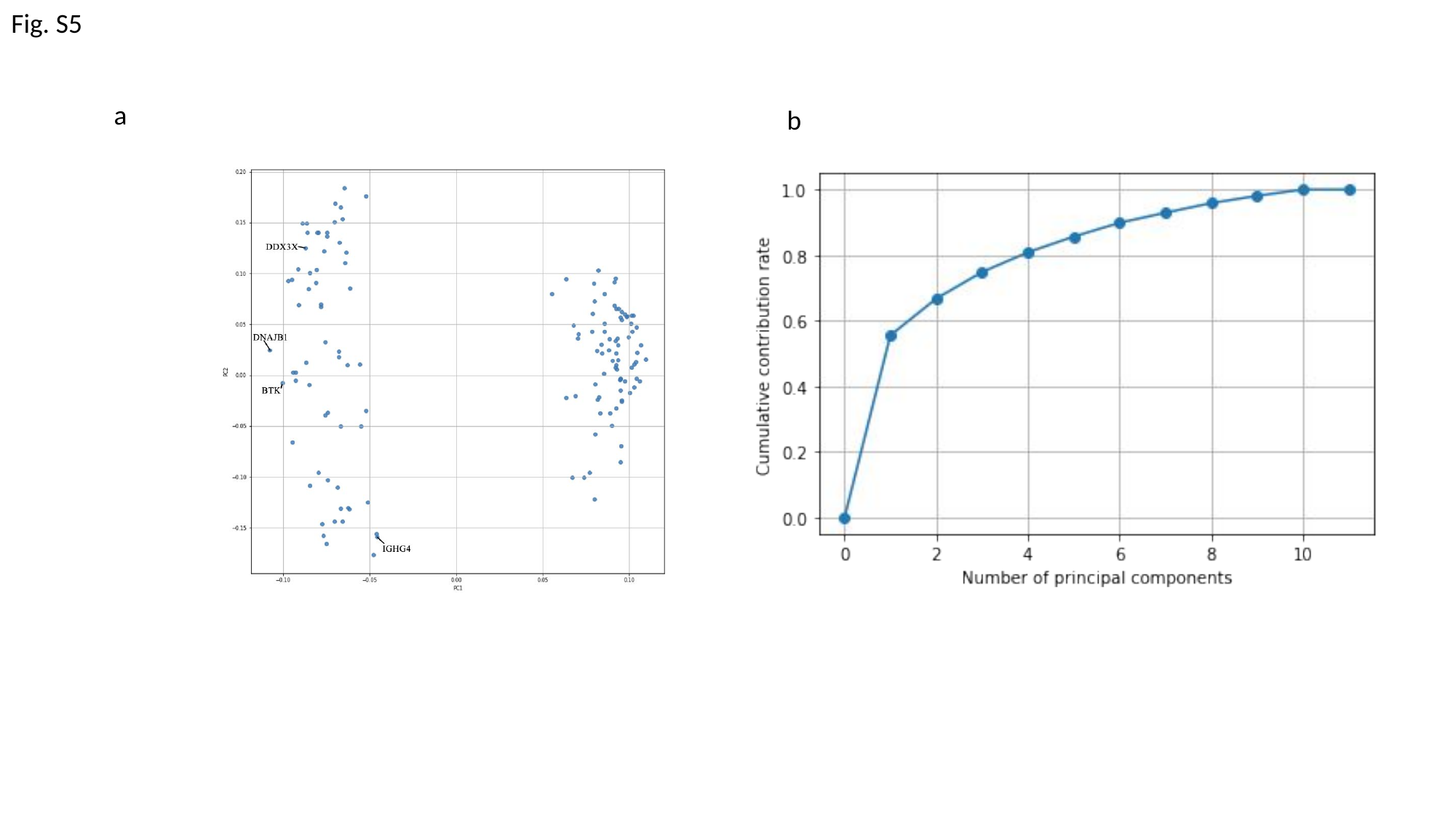

Fig. S5
a
b

## Slide 6
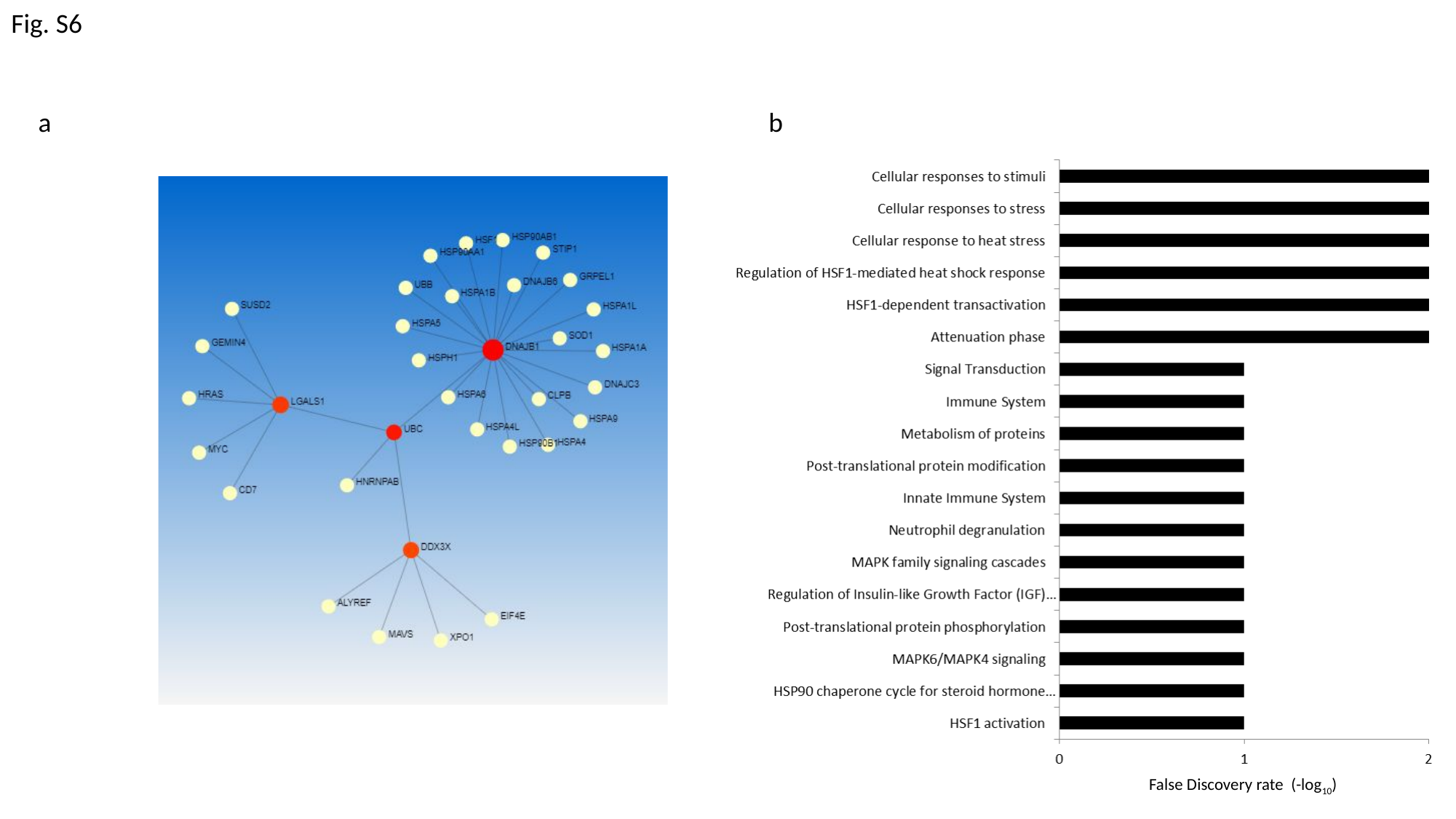

Fig. S6
a
b
False Discovery rate (-log10)
