# Supplementary material for: Proteome analysis of CD5-positive diffuse large B cell lymphoma FFPE tissue reveals downregulation of DDX3X, DNAJB1, and B cell receptor signaling pathway proteins including BTK and Immunoglobulins
Source: Clin Proteomics. 2023 Sep 13;20:36. doi: 10.1186/s12014-023-09422-z (PMC10498596; doi:10.1186/s12014-023-09422-z)
Supplement: Supplementary file 3 — Additional file 3: Table S1. Protocol for immunohistochemical assay of DLBCLs. Table S2. Correlation coefficient in patients 0–10. Table S3. Explained variance (Principal component contribution rate). Table S4. Profiling of patients of TMA. [file 12014_2023_9422_MOESM3_ESM.docx]

**Table S1. Protocol for immunohistochemical assay of DLBCLs.**

| **Ab** | **CD5** | **CD10** | **bcl6** | **MUM1** | **bcl2** | **Bruton tyrosine kinase** |
| --- | --- | --- | --- | --- | --- | --- |
| Clone | 4C7 | 56C6 | GI191E/A8 | MUM1p | 124 | HPA001198 (polyclonal) |
| Supplier | Nichirei Bioscience Inc., Tokyo, Japan | Leica Biosystems (Novocastra), Wetzlar, Germany | Roche Diagnostics, Rotkreuz, Switzerland | Agilent (DAKO), Santa Clara, CA | Agilent (DAKO), Santa Clara, CA | Atlas Antibodies, AB |
| Dilution | x1 (RTU) | x100 | x1 (RTU) | x50 | x50 | x25 |
| Reaction | 30 min, RT | 40 min, RT | 32 min, RT | 30 min, RT | 40 min, RT | 60 min, RT |
| Antigen Retrieval | Heat Processor solution ph9  Nichirei Biosciences Inc. (40 min) | Heat Processor solution pH 9 (40 min) | Ventana, BenchMark ULTRA, Ventana, Roche CC1 solution  (Std 64 min) | Heat Processor solution pH 9  (40 min) | Heat Processor solution pH 9 (40 min) | Dako Retrieval solution Proteinase K solution (S3020, Agilent, Santa Clara, CA) |
| System | Simple Stain MAX-PO (Multi) | Simple Stain MAX-PO (Multi) | Ultraview | Simple Stain MAX-PO (Multi) | Simple Stain MAX-PO (Multi) | Simple Stain™ MAX PO (Nichirei) |
| Stainer | Histostainer 48A (Nichirei Bioscience, Inc.) | Histostainer 48A | Roche Ventana (Roche Diagnostics) | Histostainer 48A | Histostainer 48A |  |

RTU, ready to use; RT, room temperature.

**References for Table S1**

1. The Human Protein Atlas. <https://www.proteinatlas.org/ENSG00000010671-BTK/pathology>

2. Sadeghi L, Arvidsson G, Merrien M, A MW, Görgens A, Smith CIE, et al, Wright AP. Differential B-cell receptor signaling requirement for adhesion of

mantle cell lymphoma cells to stromal cells. Cancers 2020. (Basel) 12.

**Table S2. Correlation coefficient in patients 0–10 (p1–p6, CD5-negative DLBCL: p7–p11, CD5-positive DLBCL)**

|  | p1(0) | p2(1) | p3(2) | p4(3) | p5(4) | p6(5) | p7(6) | p8(7) | p9(8) | p10(9) | p11(10) |
| --- | --- | --- | --- | --- | --- | --- | --- | --- | --- | --- | --- |
| p1 | 1.000000 | 0.827430 | 0.940716 | 0.695572 | 0.981194 | 0.905192 | 0.849754 | 0.858665 | 0.971214 | 0.885217 | 0.953136 |
| p2 | 0.827430 | 1.000000 | 0.875848 | 0.934633 | 0.789704 | 0.939417 | 0.468741 | 0.480233 | 0.693137 | 0.485366 | 0.655084 |
| p3 | 0.940716 | 0.875848 | 1.000000 | 0.821068 | 0.951601 | 0.962787 | 0.753161 | 0.770805 | 0.894720 | 0.751847 | 0.876784 |
| p4 | 0.695572 | 0.934633 | 0.821068 | 1.000000 | 0.665006 | 0.893562 | 0.301004 | 0.320987 | 0.554273 | 0.312676 | 0.514263 |
| p5 | 0.981194 | 0.789704 | 0.951601 | 0.665006 | 1.000000 | 0.893349 | 0.898848 | 0.908789 | 0.974991 | 0.898732 | 0.973057 |
| p6 | 0.905192 | 0.939417 | 0.962787 | 0.893562 | 0.893349 | 1.000000 | 0.629240 | 0.643629 | 0.816524 | 0.640643 | 0.789498 |
| p7 | 0.849754 | 0.468741 | 0.753161 | 0.301004 | 0.898848 | 0.629240 | 1.000000 | 0.994650 | 0.927310 | 0.972160 | 0.955639 |
| p8 | 0.858665 | 0.480233 | 0.770805 | 0.320987 | 0.908789 | 0.643629 | 0.994650 | 1.000000 | 0.939416 | 0.970271 | 0.963576 |
| p9 | 0.971214 | 0.693137 | 0.894720 | 0.554273 | 0.974991 | 0.816524 | 0.927310 | 0.939416 | 1.000000 | 0.953062 | 0.991108 |
| p10 | 0.885217 | 0.485366 | 0.751847 | 0.312676 | 0.898732 | 0.640643 | 0.972160 | 0.970271 | 0.953062 | 1.000000 | 0.965744 |
| p11 | 0.953136 | 0.655084 | 0.876784 | 0.514263 | 0.973057 | 0.789498 | 0.955639 | 0.963576 | 0.991108 | 0.965744 | 1.000000 |

**Table S3. Explained variance (Principal component contribution rate).**

| **0** | 5.553763e-01 |
| --- | --- |
| **1** | 1.120618e-01 |
| **2** | 8.102855e-02 |
| **3** | 6.024727e-02 |
| **4** | 4.787637e-02 |
| **5** | 4.208390e-02 |
| **6** | 3.070469e-02 |
| **7** | 2.929418e-02 |
| **8** | 2.222075e-02 |
| **9** | 1.910617e-02 |
| **10** | 4.898594e-33 |

**Table S4. Profiling of patients of TMA.**

| **Patient profile** | **Number** |
| --- | --- |
| **Sex** |  |
| **F** | **33** |
| **M** | **101** |
|  | **134** |
| **Age** |  |
| **10–39** | **37** |
| **40–59** | **56** |
| **60–90** | **41** |
|  |  |
| **Anatomic site** |  |
| **Bone** | **1** |
| **Cerebellum** | **4** |
| **Colon** | **6** |
| **Ileum** | **6** |
| **Liver** | **2** |
| **Lung** | **1** |
| **Lymph node** | **51** |
| **Mesentery** | **1** |
| **Ovary** | **2** |
| **Parotid gland** | **2** |
| **Skin** | **1** |
| **Small intestine** | **23** |
| **Spleen** | **3** |
| **Testis** | **21** |
|  |  |
| **ABC** | **92** |
| **GC** | **42** |
|  |  |
| **CD5+** | **9** |
| **CD5-** | **125** |

**ABC, activated B cell type.**
